# Lysosomal Acid Lipase Deficiency: Report of Five Cases across the Age Spectrum

**DOI:** 10.1155/2018/4375434

**Published:** 2018-01-21

**Authors:** Marco Antonio Curiati, Sandra Obikawa Kyosen, Vanessa Gonçalves Pereira, Francy Reis da Silva Patrício, Ana Maria Martins

**Affiliations:** ^1^Reference Center for Inborn Errors of Metabolism (CREIM), Department of Pediatrics, Universidade Federal de São Paulo (UNIFESP), São Paulo, SP, Brazil; ^2^Laboratory for Inborn Errors of Metabolism, Universidade Federal de São Paulo (UNIFESP), São Paulo, SP, Brazil; ^3^Department of Pathology, Universidade Federal de São Paulo (UNIFESP), São Paulo, SP, Brazil

## Abstract

Lysosomal acid lipase (LAL) deficiency is an autosomal recessive lysosomal storage disorder caused by mutations in the *LIPA* gene that leads to premature organ damage and mortality. We present retrospective data from medical records of 5 Brazilian patients, showing the broad clinical spectrum of the disease.

## 1. Introduction

Lysosomal acid lipase (LAL) deficiency (cholesteryl ester storage disease/Wolman disease; Online Mendelian Inheritance in Man database #278000) is an autosomal recessive lysosomal storage disorder caused by mutations in the *LIPA* gene that reduce LAL activity [1].

Deficient activity of LAL leads to the accumulation of cholesteryl esters and triglycerides in most tissues, demonstrating the enzyme's role in cholesterol and triglyceride metabolism. LAL deficiency leads to a poorly defined continuum of symptoms, historically divided into Wolman disease (early onset), first described in 1956 [2], and cholesteryl ester storage disease (late onset) [1, 3], first described in 1963 [4].

LAL deficiency presenting in infants is a neonatal-onset, severe, and lethal form, resulting in severely affected lysosomes, due to accumulation of cholesteryl esters and triglycerides, predominantly in the liver, spleen, gut, adrenal glands, bone marrow, lymph nodes, and macrophages. Affected infants present at an early age, with vomiting, diarrhea, and massive hepatosplenomegaly. Feeding difficulties and malabsorption lead to malnutrition, growth retardation, and cachexia, which, together with the severe liver disease, contribute to the patient's early demise, generally in the first year of life [5, 6].

In other cases, LAL deficiency may present in any period of life; some patients are diagnosed in childhood, while others remain undiagnosed until adulthood [7]. The progressive accumulation of lysosomal cholesteryl esters and triglycerides leads to the characteristic liver disease, elevated serum transaminase levels, and elevated serum low-density lipoprotein cholesterol (LDL-C) concentrations, with normal to low high-density lipoprotein cholesterol (HDL-C) concentrations. Premature demise is due to liver failure and/or accelerated atherosclerotic disease secondary to the chronic hyperlipidemia [1, 2, 7].

Liver biopsies may not be pathognomonic for LAL deficiency, which may be misdiagnosed as nonalcoholic fatty liver disease, nonalcoholic steatohepatitis, or cryptogenic cirrhosis. Despite being an excellent exam for diagnosis and also for evaluating the degree of liver compromise, it is an invasive exam, with potential risk of complications such as hemorrhage, specially in patients with advanced liver disease [8]. Another important tool to help elucidate the diagnosis is the endoscopy, which can present specific findings such as disseminated lipidic accumulation or other nonspecific signs, such as aesophagus varices [9]. Diagnostic suspicion can also be confirmed by demonstrating the deficient LAL activity via biochemical tests (i.e., dried blood spot, leukocytes, and fibroblasts) and determination of mutations on the *LIPA* gene by performing gene sequencing, which can help identify patients with this autosomal recessive disease without potential complications [10]. To date, the genotype-phenotype correlation has not been established.

In 2013, Bernstein et al. [11] reviewed 135 cases of LAL deficiency in the literature, showing the clinical variability of the disease. Despite the variability, the majority of cases were recognized in infancy, early childhood, or adolescence (83% of cases presented with the first clinical manifestation prior to 12 years of age), showing the important role of the pediatrician in early detection of clinical symptoms.

In this study, we report the clinical, biochemical, and histological aspects observed across the age spectrum: 1 case in infancy and 4 pediatric cases in siblings.

## 2. Methods

### 2.1. Patients

This is a retrospective study reviewing data from medical records of patients from a Brazilian Reference Center for Inborn Errors of Metabolism, Universidade Federal de São Paulo (Institutional Review Board/Ethics Committee approval number CEP-UNIFESP 2007/11).

### 2.2. Biochemical Diagnosis

LAL enzymatic activity assay was performed in dried blood spot (DBS) samples according to the fluorimetric technique described by Hamilton et al. [12] and performed at HRG Laboratory, HIBM Research Group (Chatsworth, CA, USA), which used the reference range of 0.37–2.30 nmol/punch/h. Biochemical analysis was also performed in our laboratory—Laboratório de Erros Inatos do Metabolismo (LEIM)–UNIFESP, which used the reference range of >0.024 nmol/punch/h.

### 2.3. Pathology

All patients underwent ultrasound-guided liver biopsy in order to evaluate liver damage. PAS-D stain (periodic acid-Schiff + diastasis) was performed to differentiate lipid from glycogen deposit, and Masson trichrome was performed to evaluate presence of cirrhosis.

## 3. Results

### 3.1. Case 1

The patient is a female, index case of this family, second of five siblings from the same mother, who has a son (unaffected) and a daughter (this patient) from her first marriage, and three other sons (all affected—cases 2, 3, and 4), from her second marriage. Both marriages were nonconsanguineous unions, and both fathers are not related. The mother of cases 1, 2, 3, and 4 also has reduced activity of LAL enzyme, but her case was not presented in this report.

**Figure 1:**
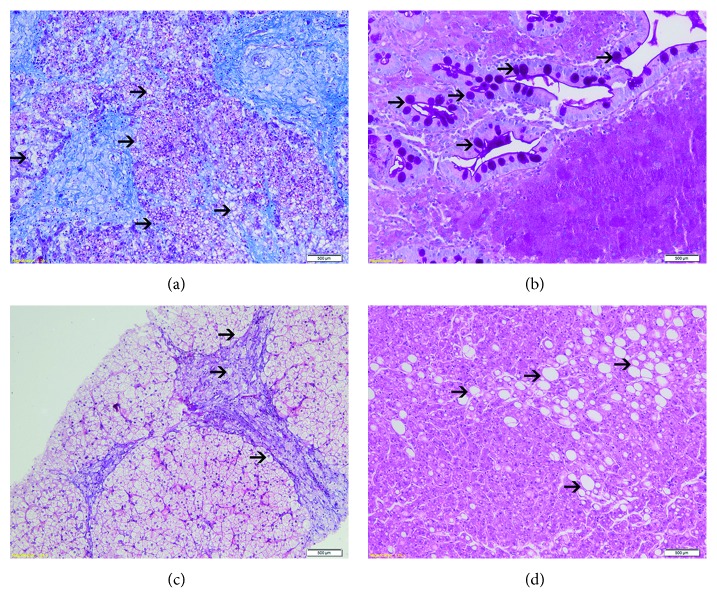
(a) Liver autopsy of case 1 showing disseminated lipid accumulation and severe steatosis with lipid-filled histiocytes (arrows) (Masson trichrome stain, 10x). (b) Duodenum biopsy of case 1 showing deformed villus, justifying the diagnosis of malabsorption syndrome. Lamina propria showing PAS+, diastasis-resistant material (arrows) (PAS+ diastase stain, 20x). (c) Liver biopsy of case 2 showing lipid-filled histiocytes and broadening portal spaces (arrows) (Masson trichrome stain, 10x). (d) Liver biopsy of case 5 showing severe macro- and microvesicular steatosis (arrows) (hematoxylin and eosin stain, 10x).

Onset of symptoms occurred at 3 years of age; she was referred to a gastroenterologist for hepatomegaly, intermittent diarrhea, and failure to thrive. She underwent liver biopsy at 3 years of age, showing granulomatous hepatitis with generalized hepatocellular stuffing. Grade 3 macro- and microvesicular diffuse steatosis was present in 60% of the hepatocytes. The patient was first evaluated in our service at the age of 4 years; she weighed 16 kg (*z* score of −0.56), her height was 93 cm (*z* score of −3.0), and she had hepatosplenomegaly (rounded liver with rock-hard consistency, 5 cm below the right costal margin; rock-hard consistency spleen, 5 cm below the left costal margin). Her second hepatic biopsy showed enlargement of portal spaces with enlarged microvacuolized macrophages and hepatocytes. She developed progressive increase of hepatosplenomegaly—by age 9, physical examination revealed that her liver was 20 cm below the right costal margin and her spleen was 17 cm below the left costal margin—and worsening of diarrheic episodes. Biochemical analysis showed dyslipidemia and mild elevation of liver transaminases (Table 1). Between 8 and 9 years of age, she underwent 7 hospitalizations due to respiratory distress secondary to severe hepatosplenomegaly. The patient died at age 9, due to pneumonia and septic shock; at that time, the enzymatic assay was not available in our country, and thus her diagnosis was confirmed by enzymatic assay in DBS samples postmortem showing LAL activity to be 0.0068 nmol/punch/h (reference range, 0.37–2.30 nmol/punch/h). The autopsy showed disseminated lipid and cholesterol crystal accumulation, mainly in the liver, spleen, bone marrow, and gastrointestinal tract, and severe steatosis with lipid-filled histiocytes (Figure 1). No adrenal calcification was noted.

### 3.2. Case 2

The patient is a male, half-brother of the patient in case 1, currently 11 years old, onset of symptoms at age 3, presenting with hepatosplenomegaly. In his first evaluation at our center at 4 years of age, clinical examination showed enlarged abdominal circumference, round liver 5 cm below the right costal margin, spleen 3 cm below the left costal margin, and growth deficit, weighing 15 kg (*z* score of −1) and measuring 96 cm (*z* score of −2.4) with a body mass index (BMI) of 16.5 (*z* score of 0.92). Laboratory assessment showed elevation in total cholesterol, with a low HDL-C level and high LDL-C level. His triglyceride level was elevated, with slight increase in liver enzymes (Table 1). Hepatic biopsy presented hepatocyte dilatation due to lipid accumulation, lipid-filled histiocytes, and broadening portal spaces suggesting lipid storage disease (Figure 1). Enzymatic assay in DBS samples showed LAL activity to be 0.0087 nmol/punch/h (reference range, 0.37–2.30 nmol/punch/h), confirming the diagnosis.

### 3.3. Case 3

The patient is a male, younger half-brother of the patient in case 1, currently 10 years old, diagnosed at the age of 6 years in the familial screening. In his first evaluation, at 6 years old, no hepatosplenomegaly and no growth delay were noticed, with the patient weighing 20 kg (*z* score of −0.46) and measuring 114 cm (*z* score of −0.77), with a BMI of 15.4 kg/m^2^ (*z* score of 0.03). Laboratory assessment showed mild elevation of total cholesterol and the LDL-C fraction, and a low HDL-C fraction. Triglycerides were also mildly elevated (Table 1), but transaminase levels were normal. Abdominal sonogram showed discrete homogeneous hepatomegaly for the patient's age. Enzymatic assay of DBS samples showed LAL activity to be 0.00 nmol/punch/h (reference range, >0.024 nmol/punch/h), confirming the diagnosis.

### 3.4. Case 4

The patient is a male, younger half-brother of the patient in case 1 currently 8 years old, who was diagnosed at the age of 3 years in the familial screening. In his first assessment in our service, at the age of 2 years and 11 months, the patient presented with sporadic episodes of diarrhea. No hepatomegaly and no growth delay were noticed; the patient weighed 16 kg (*z* score of 0.9) and measured 95.5 cm (*z* score of −0.35), with a BMI of 17.8 kg/m^2^ (*z* score of 1.63). Laboratory assessment showed that his total cholesterol level was within the normal range, but he had mildly low HDL-C and mildly high LDL-C levels. Triglycerides were also elevated (Table 1). Abdominal sonogram showed that the liver and spleen were within the upper limit of normal for the patient's age. Enzymatic assay of DBS samples showed LAL activity to be 0.00 nmol/punch/h (reference range, >0.024 nmol/punch/h), confirming the diagnosis.

### 3.5. Case 5

The patient is a male, not related to the previous patients, with neonatal cholestasis until 20 days of life. He was first evaluated at our center in 2012 at 4 months of age with a perinatal history of cholestasis and hypoglycemia; severe jaundice (Kramer scale score of 5); severe hepatosplenomegaly (liver and spleen 5 cm below the costal margin); failure to thrive, weighing 5150 g (*z* score of −2.51) and measuring 60.5 cm (*z* score of −1.43), with a BMI of 14.1 (*z* score of −2.35); blood dyscrasia; impaired liver function, with severe hepatic and canalicular enzyme level elevations; and very high total cholesterol, low HDL-C, and high LDL-C levels. Triglycerides were also elevated (Table 1). The patient presented with severe pancytopenia and hemophagocytic lymphohistiocytosis syndrome. By the age of 8 months, the patient underwent liver transplantation due to hepatic failure and died soon after the procedure due to acute organ rejection. Liver biopsy showed severe macro- and microvesicular steatosis with associated severe fibrosis and portal ductular reaction (Figure 1). The DBS samples showed LAL activity to be 0.0324 nmol/punch/h (reference range, 0.37–2.30 nmol/punch/h). *LIPA* gene sequencing presented two heterozygous mutations: an exon 5 deletion (c.477delT) and an exon 7 insertion (c.822+37_38insC).

## 4. Discussion

Clinically, LAL deficiency, like other lysosomal storage disorders, can present across the age spectrum. It is often unrecognized in infants as well as in children and adults. In children and adults, once a physician notes the hepatomegaly, with or without splenomegaly and/or elevated transaminase activity, suspicion for LAL deficiency increases, but this first requires an awareness of the disease [4]. Typically, a liver biopsy will reveal foamy, lipid-filled hepatocytes and macrophages, microvesicular steatosis, and lysosomal cholesteryl ester crystals, best observed by ultrastructural examination [13].

The hepatosplenomegaly in cases 1 and 2 was noticed by 3 years of age; this finding can be an alert to pediatricians and hepatologists, once frequent causes of hepatomegaly are discarded, with or without splenomegaly, as reported by Bernstein et al. [11] in 2013, after analyzing 135 cases of LAL deficiency found that 99,3% of the patients presented hepatomegaly and 74% presented splenomegaly, being those the most frequent first signs of the disease. In contrast, the patient in case 3 did not have evidence of hepatomegaly at age 6, demonstrating the variety of the clinical spectrum, even between siblings, who are supposed to carry the same mutation. On the other hand, the patient in case 5 presented at an early age with severe hepatosplenomegaly and early-onset hepatic failure. When compared to the other cases, the patient in case 5 presented a much more severe and much more early-onset symptoms, showing the clinical variability of this disease.

Regarding the dyslipidemia, four out of five patients showed abnormal levels of total cholesterol, and all patients showed abnormal cholesterol fractions and triglycerides, being higher on the patient with an early-onset “Wolman” phenotype. In children and adults with LAL deficiency, laboratory parameters of cholesterol are not always severely abnormal, as reported by Bernstein et al. in 2013, when reviewing 135 cases of patients with LAL deficiency, where 79% of the patients presented total cholesterol > 200 and 71% had HDL-C between 20 and 40 [11]. As previously reported, patients with higher levels of total cholesterol and LDL-C do not necessarily present with more severe lipid deposits and impaired liver function than patients with lower levels [7].

Regarding liver enzymes, we can observe that patients 1 to 4 had moderately increased aspartate aminotransferase, alanine aminotransferase, gamma-glutamyltransferase, and alkaline phosphatase levels, and patient 5 showed much more severe compromise of liver function, with much higher enzyme levels.

The liver biopsies of the patients in cases 1 and 2 show us disseminated lipid accumulation and severe steatosis with lipid-filled histiocytes, marking the important lipid deposits in hepatocytes, leading to cellular damage and subsequent organic failure. In case 5, we can see more important tissue damage, with severe macro- and microvesicular steatosis and fibrosis presenting at an early stage. That follows the pattern showed in the literature so far [14, 15] and, despite the initial presenting symptoms or signs, the presence of dyslipidemia, hypertriglyceridemia, and increased liver enzyme levels must lead to the suspicion of LAL deficiency.

These cases in addition to the published literature [1, 16–18] show the broad clinical spectrum of this disease and highlight the importance of health care professionals having LAL deficiency in mind as a potential diagnosis when evaluating a patient with hepatomegaly with or without splenomegaly, elevated liver enzymes, dyslipidemia, and/or growth abnormalities, because the early identification of patients is an important prerequisite for timely treatment with enzyme replacement therapy [19].

## 5. Conclusion

LAL deficiency should be included in the differential diagnosis for all patients with elevated LDL-C who also may have mildly to moderately decreased HDL-C levels, elevated transaminases, and hepatomegaly. Enzyme replacement therapy with sebelipase alfa is an option in some areas of the world. Thus, the early detection of patients is advised, because the earlier the patient starts the treatment, the better the outcome will probably be.

## Figures and Tables

**Table 1:** Laboratory assessment of patients at the time of diagnosis.

	Patient 1^a^	Patient 2	Patient 3	Patient 4	Patient 5	Reference range
AST (U/L)	55	80	35	39	909	<40
ALT (U/L)	35	76	30	38	1449	<41
GGT (U/L)	17	19	19	17	754	≤60
Alkaline phosphatase (U/L)	194	140	243	221	668	<390
Total cholesterol (mg/dL)	212	222	177	164	287	<170
HDL-C (mg/dL)	12	19	34	31	28	>39
LDL-C (mg/dL)	148.8	170	146	117	234	<110
VLDL-C (mg/dL)	51.2	33	—	—	25	—
Triglycerides (mg/dL)	256	167	108	111	125	<100
Total bilirubin (mg/dL)	4.04	0.5	0.39	0.7	16.08	<1
DB (mg/dL)	3.2	0.2	0.13	0.2	12.64	0.0–0.2
IB (mg/dL)	0.84	0.3	0.26	0.5	3.44	0.1–0.6

ALT, alanine aminotransferase; AST, aspartate aminotransferase; DB, direct bilirubin; GGT, gamma-glutamyltransferase; HDL-C, high-density lipoprotein cholesterol; IB, indirect bilirubin; LDL-C, low-density lipoprotein cholesterol; VLDL-C, very-low-density lipoprotein cholesterol. ^a^Samples were collected by the time the clinical diagnosis of LALD was suspected, since the biochemical diagnosis was established postmortem.

## Abbreviations

BMI: Body mass index
HDL-C: High-density lipoprotein cholesterol
LAL: Lysosomal acid lipase
LDL-C: Low-density lipoprotein cholesterol
*LIPA*: Lysosomal acid lipase gene
PAS: Periodic acid-Schiff
DBS: Dried Blood Spots.
